# Golgi tethering factor golgin-97 suppresses breast cancer cell invasiveness by modulating NF-κB activity

**DOI:** 10.1186/s12964-018-0230-5

**Published:** 2018-04-27

**Authors:** Rae-Mann Hsu, Cai-Yan Zhong, Chih-Liang Wang, Wei-Chao Liao, Chi Yang, Shih-Yu Lin, Jia-Wei Lin, Hsiao-Yun Cheng, Po-Yu Li, Chia-Jung Yu

**Affiliations:** 1 Department of Cell and Molecular Biology, College of Medicine, Chang Gung University, Taoyuan, Taiwan; 2 Graduate Institute of Biomedical Sciences, College of Medicine, Chang Gung University, Taoyuan, Taiwan; 3 School of Medicine, College of Medicine, Chang Gung University, Taoyuan, Taiwan; 4 Division of Pulmonary Oncology and Interventional Bronchoscopy, Department of Thoracic Medicine, Chang Gung Memorial Hospital, Linkou, Taoyuan, Taiwan; 5 Molecular Medicine Research Center, Chang Gung University, Taoyuan, Taiwan; 6 Department of Otolaryngology - Head & Neck Surgery, Chang Gung Memorial Hospital, Linkou, Taoyuan, Taiwan; 7 Center for General Education, Chang Gung University, Taoyuan, Taiwan

**Keywords:** Golgin-97, TGN, Golgi apparatus, Cell migration, NF-κB, Breast cancer

## Abstract

**Background:**

Golgin-97 is a tethering factor in the *trans*-Golgi network (TGN) and is crucial for vesicular trafficking and maintaining cell polarity. However, the significance of golgin-97 in human diseases such as cancer remains unclear.

**Methods:**

We searched for a potential role of golgin-97 in cancers using Kaplan-Meier Plotter (http://kmplot.com) and Oncomine (www.oncomine.org) datasets. Specific functions of golgin-97 in migration and invasion were examined in golgin-97-knockdown and golgin-97-overexpressing cells. cDNA microarray, pathway analysis and qPCR were used to identify gene profiles regulated by golgin-97. The role of golgin-97 in NF-κB signaling pathway was examined by using subcellular fractionation, luciferase reporter assay, western blot analysis and immunofluorescence assay (IFA).

**Results:**

We found that low expression of golgin-97 correlated with poor overall survival of cancer patients and was associated with invasiveness in breast cancer cells. Golgin-97 knockdown promoted cell migration and invasion, whereas re-expression of golgin-97 restored the above phenotypes in breast cancer cells. Microarray and pathway analyses revealed that golgin-97 knockdown induced the expression of several invasion-promoting genes that were transcriptionally regulated by NF-κB p65. Mechanistically, golgin-97 knockdown significantly reduced IκBα protein levels and activated NF-κB, whereas neither IκBα levels nor NF-κB activity was changed in TGN46- or GCC185-knockdown cells. Conversely, golgin-97 overexpression suppressed NF-κB activity and restored the levels of IκBα in golgin-97-knockdown cells. Interestingly, the results of Golgi-disturbing agent treatment revealed that the loss of Golgi integrity was not involved in the NF-κB activation induced by golgin-97 knockdown. Moreover, both TGN-bound and cytosolic golgin-97 inhibited NF-κB activation, indicating that golgin-97 functions as an NF-κB suppressor regardless of its subcellular localization.

**Conclusion:**

Our results collectively demonstrate a novel and suppressive role of golgin-97 in cancer invasiveness. We also provide a new avenue for exploring the relationship between the TGN, golgin-97 and NF-κB signaling in tumor progression.

**Electronic supplementary material:**

The online version of this article (10.1186/s12964-018-0230-5) contains supplementary material, which is available to authorized users.

## Background

The *trans*-Golgi network (TGN) is a major traffic hub of the cell where newly synthesized proteins are sorted from the Golgi apparatus via anterograde trafficking (in to out), and endocytic or recycling cargos are transported into the Golgi by retrograde transport (out to in). It is increasingly clear that the Golgi functions in several biological processes including cytoskeletal dynamics, organelle biogenesis, receptor signaling, apoptosis and mitosis [1–3]. Golgins, a family of coiled-coil proteins associated with the Golgi apparatus, are required for tethering events in membrane fusion and maintaining the Golgi architecture [2, 4–8]. There are four GRIP (**g**olgin-97/**R**anBP2α/**I**mh1/**p**230/golgin-245)-domain golgins in mammalian cells, including golgin-97, golgin-245/p230, GCC185, and GCC88, whereas Imh1 is the only GRIP-domain golgin in yeast [9–11]. The four mammalian GRIP domain proteins differ in their membrane-binding properties and are recruited to distinct domains of the TGN [12]. Both golgin-97 and golgin-245 act as scaffold molecules and are recruited onto the TGN by interacting with Arf-like protein 1 (Arl1), an Arf small GTPase family member in the Ras superfamily, to establish a molecular platform required for numerous distinct processes of vesicle transport [2, 6–8, 13–15]. For example, golgin-245 was identified as an essential regulator of tumor necrosis factor (TNF)-α and interleukin-6 secretion in vitro and in vivo [16–18]. However, the roles of these GRIP domain golgins in human diseases remain unclear.

In 1997, golgin-97 was the fifth unique Golgi complex autoantigen to be cloned and characterized in a study identifying autoantigens in sera from patients with Sjogren’s syndrome [19]. Golgin-97 plays essential roles as a tethering molecule associated with tubulovesicular carriers during vesicle trafficking and in maintaining the Golgi integrity [2, 4, 5, 8, 12, 17, 20]. Depletion of golgin-97 via microinjection of an anti-golgin-97 antibody into HeLa cells impaired transport of Shiga toxin subunit B from early endosomes to the TGN [21], and knockdown of golgin-97 in HeLa cells blocked the exit of green fluorescent protein (GFP)-tagged E-cadherin cargo from the TGN [16]. Considering that E-cadherin forms cell-cell adherens junctions, which are crucial for tissue integrity and epithelial-to-mesenchymal transition (EMT) during tumor metastasis, we postulated that golgin-97 might play a role in modulating tumorigenesis.

In this study, we investigated the potential pathological role of golgin-97 in cancers via Kaplan-Meier Plotter (http://kmplot.com/analysis/) and Oncomine (https://www.oncomine.org/resource/login.html) databases, and we proposed that golgin-97 acts as a tumor suppressor. Using a breast cancer cell line as a model, we demonstrated that golgin-97 knockdown caused a reduction in IκBα levels and an increase in NF-κB activity, which then promoted cell migration and invasion. We also observed that loss in Golgi integrity did not seem to be involved in the NF-κB activation induced by golgin-97 knockdown. Interestingly, we found that both TGN-bound and cytosolic golgin-97 suppress cell motility and NF-κB activity. Our results collectively uncover a novel role of golgin-97 in suppressing cancer invasiveness and provide potential targets for cancer treatments.

## Methods

### Kaplan-Meier plotter analysis

The prognostic value of the golgin-97 gene in human cancers was analyzed using Kaplan-Meier Plotter (http://kmplot.com/analysis/), a database that integrates gene expression and clinical data. To date, Kaplan-Meier Plotter contains information on 54,675 genes and their effect on survival in 5143 breast, 1816 ovarian, 2437 lung and 1065 gastric cancer patients, with a mean follow-up of 69, 40, 49 and 33 months, respectively. Patient samples were split into two groups (high and low expression levels) based on a median value of golgin-97 gene expression. Overall survival was analyzed by using a Kaplan-Meier survival plot with an auto-select best cutoff.

### Analysis of golgin-97 expression levels from tissue transcriptome datasets

The Oncomine database (https://www.oncomine.org/resource/login.html), a cancer microarray database and web-based data-mining platform, was used to search golgin-97 mRNA expression profiles in breast and lung cancer. The Richardson Breast 2 dataset contains differential gene profiles generated from 7 breast and 40 ductal breast carcinoma tissues [22]. The Hou Lung dataset contains differential gene profiles generated from 65 lung and 19 large cell lung carcinoma tissues [23]. The Radvanyi Breast dataset contains differential gene profiles generated from 2 ductal breast carcinomas in situ and 18 invasive ductal breast carcinoma tissues [24]. Box plots of these three studies compared the mRNA levels of golgin-97 in normal tissues and cancer tissues or the levels in cancer tissues with different pathological stages. A gene with a 1.5-fold change in expression with a *p*-value less than 0.05 was considered differentially expressed.

### Cell culture and transfection

HeLa, MCF7, MDA-MB-157, MDA-MB-468 and MDA-MB-231 cells were maintained in Dulbecco’s Modified Eagle’s Medium (Gibco, Invitrogen, Carlsbad, CA, USA) supplemented with 10% FBS and antibiotics and cultured at 37 °C in a humidified atmosphere of 95% air/5% CO_2_. T47D cells were maintained in RPMI Medium (Gibco, Invitrogen, Carlsbad, CA, USA) supplemented with 10% FBS and antibiotics and cultured at 37 °C in a humidified atmosphere of 95% air/5% CO_2_. For gene knockdown, cells were transfected with control, golgin-97, TGN46 or GCC185 Ambion siRNA (Thermo Fisher Scientific, USA) (sequences were listed in Additional file 1: Table S1) using Lipofectamine RNAiMAX reagents (Invitrogen, Grand Island, NY, USA) for 48 h, according to the manufacturer’s instructions. For rescue experiments, indicated plasmids were transfected into golgin-97-knockdown MDA-MB-231 cells using Lipofectamine 2000 (Invitrogen, Grand Island, NY, USA) for 24 h, according to the manufacturer’s instructions.

### Plasmid constructs

Human golgin-97 cDNA from HeLa cells was PCR-amplified and cloned into pEGFP-C1 and pEGFP-N3 vectors (Clontech, Mountain View, CA, USA) to generate N-terminal EGFP-tagged and tag-free golgin-97-expressing plasmids, respectively. We replaced the codon for Tyr697 with the codon for Ala to generate an Arl1-binding-defective mutant (Y697A) [25]. We deleted the entire GRIP domain (amino acids 688–737) and the carboxyl terminus (amino acids 688–767) of golgin-97 to generate del-GRIP and del-C mutants, respectively. Plasmids containing golgin-97 wild-type (WT), Y697A (YA), del-GRIP and del-C with resistance to golgin-97 siRNA-1 were generated by using a QuikChange Multi Site-Directed Mutagenesis Kit (Agilent Technologies, Stratagene Products Division, La Jolla, CA, USA), according to the manufacturer’s instructions. Primer sequences are provided in Additional file 2: Table S2.

### Immunoprecipitation

To examine the interaction between golgin-97 and Arl1, HeLa cells expressing EGFP-golgin-97 WT or the YA mutant were extracted by incubation in NS buffer (20 mM HEPES, pH 7.5, 100 mM NaCl, 5 mM MgCl_2_, 1 mM DTT) with 10 μM GTPγS and were subsequently immunoprecipitated using GFP-Trap magnetic agarose beads from ChromoTek GmbH (Martinsried, Germany) according to the manufacturer’s instructions. Input (30 μg) and IP products were resolved by SDS-PAGE followed by western blot with the indicated antibodies.

### Harvesting conditioned medium from cancer cell lines

Conditioned medium (CM) from control or golgin-97-knockdown MDA-MB-231 cells was collected as previously described [26]. Briefly, cells were grown to confluence in tissue culture dishes, washed with serum-free medium, and incubated in serum-free medium for 24 h. Supernatants were then collected, concentrated and desalted. Protein concentrations of collected supernatants were determined using a BCA protein assay from Pierce (Rockford, IL, USA).

### Transwell migration and invasion assay

Cell migration was assessed using a 24-well transwell chamber (8.0-μm pore size filter; Corning, Canton, NY). Cells transfected with control or golgin-97 siRNA were harvested and resuspended in serum-free culture medium. The cell suspension (200 μl; 7.5 × 10^4^ cells) was added to each upper chamber, and each lower chamber was filled with 600 μl of serum-free culture medium containing 10 μl/ml fibronectin. After a 6-h incubation at 37 °C, chambers were gently washed twice with PBS, methanol fixed and then Giemsa stained. Cells that had traversed the filter to the lower chamber were counted microscopically (400×) in six different fields per filter. For the invasion assay, the cell suspension was added to Matrigel Matrix (Corning, Canton, NY, USA)-pre-coated transwells and incubated for 24 h at 37 °C, and the assay was conducted using the same procedure as the migration assay.

### Immunofluorescence assay (IFA)

Transfected cells were cultured on glass coverslips, fixed with 4% formaldehyde and treated with permeabilized buffer (0.1% Triton X-100 and 0.05% SDS in PBS). Cells were then blocked in blocking solution (0.1% saponin, 0.2% bovine serum albumin in PBS) and incubated with primary antibodies. After washing, cells were further incubated with Alexa Fluor 594-conjugated or Alexa Fluor 488-conjugated secondary antibodies for 1 h (Molecular Probes, Eugene, OR, USA), and nuclei were stained with Hoechst 33,258. Following a second wash with PBS, cells were mounted with 90% glycerol in PBS containing 1 mg/ml of ρ-phenylenediamine and observed under a Zeiss Axio Imager Z1 microscope (Carl Zeiss, Gottingen, Germany). Images were acquired using a Zeiss ApoTome fluorescence microscope and AxioVision Rel 4.8 software.

### Microarray analysis of gene expression

Total RNA isolated from control and golgin-97-knockdown MDA-MB-231 cells was prepared and subjected to hybridization on the Agilent SurePrint G3 Human V2 GE 8 × 60 K Microarray (Agilent Technologies, CA, USA) according to the manufacturer’s protocol. Microarray data were normalized according to the median intensity of each sample. We applied a 2-fold change as the criterion to select differentially expressed genes upon golgin-97 knockdown.

### Quantitative reverse transcription polymerase chain reaction (RT-qPCR)

Total RNA was extracted from control or golgin-97 siRNA-transfected MDA-MB-231 cells using TRIzol reagent (Invitrogen, Grand Island, NY, USA) and was synthesized via RT-PCR with a ToolsQuant II Fast RT kit (TOOLS Biotechnology Co., Ltd. Taiwan). Real-time quantitative PCR (qPCR) was performed with a 20-μl reaction mixture containing 750 nM forward and reverse primers, varying amounts of template and 1× Power SYBR Green reaction mix (Applied Biosystems, Foster City, CA). Primers are listed in Additional file 2: Table S2. SYBR Green fluorescence intensity was determined using an ABI PRISM 7500 detection system (Applied Biosystems), and expression levels of specific genes were normalized against those of β-actin, which was used as a control gene.

### Pathway enrichment analysis

Differentially expressed genes (DEGs) identified upon golgin-97 knockdown (more than a 2-fold change) were subjected to pathway analysis using the Kyoto Encyclopedia of Genes and Genomes (KEGG) database (http://www.genome.jp/kegg/). To analyze transcriptionally regulated pathways, these differentially expressed genes were uploaded to MetaCore software (GeneGo, St. Joseph, MI) and analyzed using network (transcription factors) algorithms according to the manufacturer’s instructions.

### Transcription factor enrichment analysis

To systematically analyze the transcriptional regulations in the golgin-97-knockdown cells, a promoter analysis for the 428 upregulated genes upon golgin-97 knockdown was analyzed by using the TRRUST v2 database [27], a manually curated database that collects transcription factor (TF)-target regulatory relationships based on experimental observations rather than bioinformatics prediction. A *p*-value less than 0.05 determined by Fisher’s exact test was used to indicate the significance of the TF enrichment.

### Subcellular fractionation

Cells were subjected to subcellular fractionation using NE-PER Nuclear and Cytoplasmic Extraction Reagents (Thermo Fisher Scientific, Lafayette, CO, USA), according to the manufacturer’s instructions. Fractionation efficacy was determined via western blot analysis using GAPDH as a cytosolic control and Lamin A/C as a nuclear control protein.

### Reagents and antibodies

NF-κB inhibitor BAY 11–7085 (SC-202490, Santa Cruz Biotechnology, Dallas, TX, USA) and monensin (M5273, Sigma Chemical Co., St. Louis, MO, USA) were dissolved in dimethyl sulfoxide (DMSO) and diluted in fresh medium before use. Antibodies used in this study and their sources are as follows: anti-golgin-97 (ab84340; Abcam Inc., Cambridge, MA, USA); anti-E-cadherin (sc-7870), anti-p65 (sc-8008), anti-GAPDH (sc-6215), and anti-Lamin A/C (sc-6215) (Santa Cruz Biotechnology, Dallas, TX, USA); anti-IκB (#4814; Cell Signaling Technology, Beverly, MA, USA); anti-phospho-NF-κB p65 (Ser529) (GTX38622; GeneTex, Irvine, CA, USA); anti-β-actin (Millipore, Bedford, MA, USA); anti-TGN46 (AD Serotec, AHP500; Bio-Rad Laboratories, Inc., Hercules, CA, USA); and anti-GM130 (610823; BD Transduction Laboratories, Lexington, KY, USA). The rabbit polyclonal antibody against human Arl1 was produced in-house as described previously [28]. Anti-GCC185 was kindly provided by Dr. Fang-Jen S. Lee, National Taiwan University, Taiwan.

### Luciferase assay

Cells were transfected with NF-κB-responsive reporter p1242 3×-κB-Luc (https://www.addgene.org/26699/) and a Renilla luciferase plasmid for 24 h, and then, cell lysates were prepared, collected and assayed using a dual luciferase assay system (Promega Corp., Madison, WI) according to the manufacturer’s instructions. Firefly luciferase activity derived from plasmid p1242 3×-κB-Luc was normalized to its respective Renilla luciferase activity as a control for the transfection efficiency. The results are expressed as fold induction relative to the basal levels measured in cells transfected with control or golgin-97 siRNA. Values are presented as the mean ± SEM (mean of standard deviation) from three independent transfections performed in parallel and are representative of at least three experiments.

### Statistical analysis

All quantitative data are shown as the mean ± SEM (mean of standard deviation). Significant differences between two groups were assessed by two-sided t test or ANOVA using Graph Pad Prism 6.02 software.

## Results

### Low expression of golgin-97 correlates with poor overall survival of cancer patients and is associated with invasiveness in breast cancer cells

**Fig. 1:**
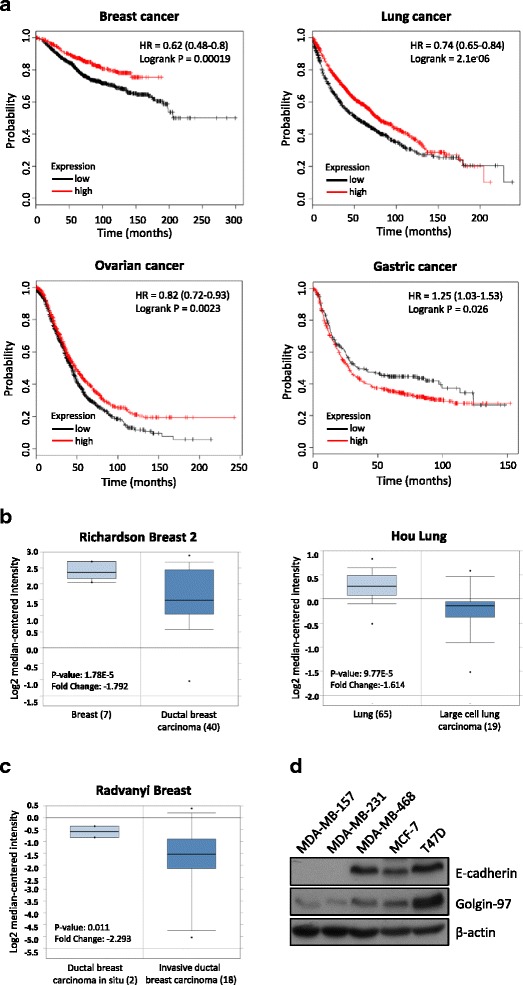
Golgin-97 expression correlates with the overall survival of cancer patients and breast cancer cell invasiveness. **a** Survival curves from Kaplan-Meier plot profiles for cancer patients stratified by high and low expression of golgin-97 (203384_s_at reporter on Affymetrix GeneChip). **b** Box plots derived from gene expression data from Oncomine that compare expression of golgin-97 mRNA levels in ductal breast carcinoma and large cell lung carcinoma with the levels in normal tissues. **c** Comparison of golgin-97 mRNA levels in tissue samples from ductal breast carcinoma in situ and invasive ductal breast carcinoma. **d** Western blot analysis of golgin-97 and E-cadherin expressions in five breast cell lines with different invasiveness potentials. Actin was used as the internal control

The effect of golgin-97 gene expression on survival of four human cancer types was analyzed according to the Kaplan-Meier Plotter database (http://kmplot.com/analysis/), and Fig. 1a shows that low expression of golgin-97 positively correlated with a poorer overall survival in breast (*n* = 1083), lung (*n* = 828) and ovarian (*n* = 1656) cancer patients. Based on the Oncomine database, we also found that golgin-97 mRNA levels in ductal breast carcinoma and large cell lung carcinoma were significantly lower than those in normal tissues (Fig. 1b, 1.5-fold change with a *p*-value less than 0.05). Notably, the Radvanyi Breast dataset showed that the golgin-97 mRNA levels in invasive ductal breast carcinoma were lower (2.293-fold change with a *p*-value = 0.011) than those in ductal breast carcinoma in situ (Fig. 1c). Based on the molecular features and morphological characteristics of different subtypes of breast cancer cell lines published recently [29], we evaluated golgin-97 expression in five breast cell lines with different invasiveness potentials (MDA-MB-157 and MDA-MB-231 > MDA-MB-468 > MCF-7 and T47D) to further elucidate whether golgin-97 expression negatively correlates with breast cancer invasiveness. Figure 1d shows that golgin-97 protein levels in more invasive breast cancer cell lines (MDA-MB-157 or MDA-MB-231) were lower than those in less invasive cell lines (MDA-MB-468, MCF-7, and T47D). We also observed that golgin-97 knockdown reduced the levels of cell surface-bound E-cadherin in breast cancer cells (Additional file 3: Figure S1). These results collectively suggest that downregulation of golgin-97 is positively associated with poor prognosis and cancer invasiveness in breast cancer.

### Golgin-97 knockdown promotes breast cancer cell motility

**Fig. 2:**
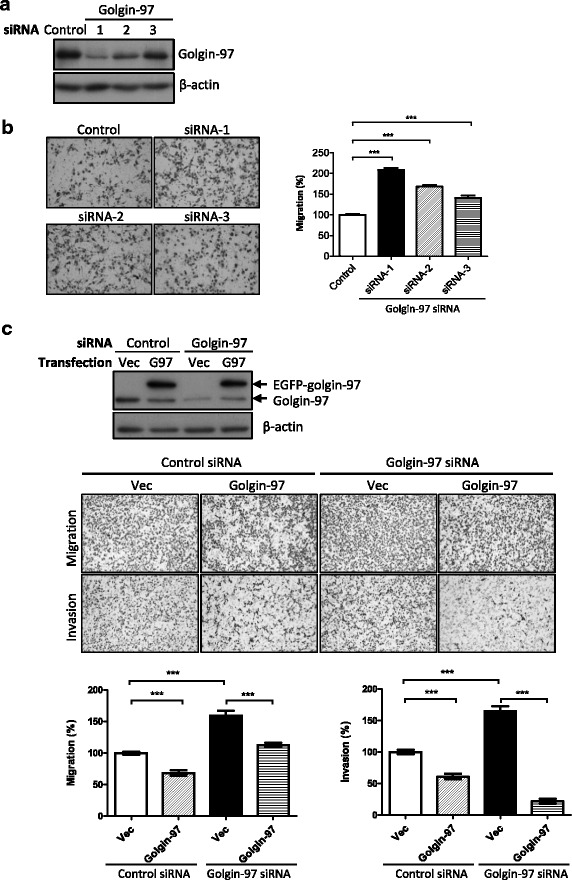
Golgin-97 knockdown promotes cell migration and cell invasion. **a** Western blot analysis of golgin-97 expression in MDA-MB-231 cells transfected with control or golgin-97-specific siRNA, respectively. Actin was used as the internal control. **b** Knockdown of golgin-97 induces cell migration and invasion. **c** Re-expression of golgin-97 inhibits golgin-97 knockdown-induced cell migration and invasion. Quantitative analysis of migration and invasion assays is presented as the mean ± SEM obtained from three independent experiments. ***p < 0.001 (**b** and **c**)

To clarify the role of golgin-97 in tumorigenesis, we used siRNA oligos to knock down golgin-97 gene expression in breast cancer cells and then examined the effects on cell viability, migration and invasion. Knockdown efficiency of individual golgin-97 siRNAs (siRNA-1, − 2, or − 3) in MDA-MB-231 cells was analyzed by western blot. Notably, protein levels of golgin-97 in siRNA-1 oligo-transfected cells were significantly reduced to approximately 20% of the levels in control oligo-transfected cells (Fig. 2a). Simultaneously, results of the transwell migration assay indicated that the cell migration abilities were significantly increased to 208%, 168% and 141% in golgin-97 siRNA-1-, siRNA-2- and siRNA-3-transfected cells, respectively, compared with control siRNA-transfected cells (Fig. 2b). However, golgin-97 knockdown had no effect on cell viability or growth (data not shown). To determine whether re-expression of golgin-97 in golgin-97-knockdown cells can restore the migration ability, we generated a golgin-97 siRNA-1-resistant construct and then performed rescue experiments. As expected, overexpression of the golgin-97 siRNA-1-resistant construct indeed suppressed golgin-97 knockdown-induced cell migration and invasion (Fig. 2c). In addition, compared with the mock (vector) control, we found that overexpression of golgin-97 in control siRNA-transfected cells also caused a significant reduction in cell migration and invasion (Fig. 2c). Taken together, these results demonstrated a suppressive role of golgin-97 in regulating breast cancer cell migration and invasion.

### NF-κB signaling pathway is involved in golgin-97-mediated migration and invasion of cancer cells

**Fig. 3:**
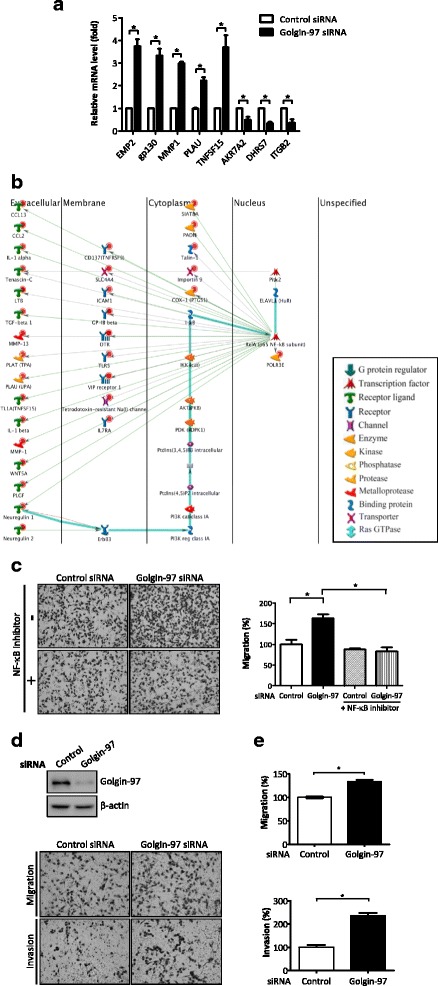
NF-κB activation is involved in golgin-97-mediated cell migration and invasion. **a** qPCR analysis of the indicated significantly regulated genes in golgin-97-knockdown cells. The results are expressed as the mean ± SEM from triplicate experiments. *p < 0.05. **b** NF-κB p65 controls the significant network deduced from the 428 upregulated genes identified from the comparison between golgin-97-knockdown cells and control cells. Nodes represent gene names, and lines between nodes indicate protein-protein interactions, with green, red, and gray lines denoting positive, negative, and unspecified effects, respectively. Highlighted lines indicate canonical pathways. Global nodes highlight genes identified in the current study, and red bottoms indicate upregulated genes in golgin-97-knockdown cells. **c** Treatment with NF-κB inhibitor abolishes golgin-97-knockdown-induced cell migration. Representative microphotographs of filters were obtained from the migration/invasion assays. Original magnification: 100×. *p < 0.05. **d** Treatment of CM derived from golgin-97-knockdown cells promoted cell migration/invasion abilities of recipient cells. Western blot analysis was used to confirm the knockdown efficacy. Representative microphotographs of filters were obtained from the migration/invasion assays. Original magnification: 100×. **e** Quantitative analysis of the migration/invasion assays is presented as the mean ± SEM obtained from three independent experiments. *p < 0.05

To systematically investigate golgin-97-associated biological networks, global gene expression profiling of control and golgin-97-knockdown MDA-MB-231 cells was performed by cDNA microarray analysis. Of the differentially expressed genes with 2-fold changes affected by golgin-97 knockdown, 428 were found to be significantly upregulated, and 511 were downregulated. To validate the credibility of this result, we used qPCR to verify the following eight candidates randomly selected from the differentially expressed genes (DEGs): five upregulated genes, epithelial membrane protein 2 (EMP2), glycoprotein 130 (gp130), matrix metalloproteinase-1 (MMP1), urokinase-plasminogen activator (PLAU), and tumor necrosis factor super family 15 (TNSF15); and three down-regulated candidates, aldo-keto reductase family 7 member A2 (AKR7A2), dehydrogenase/reductase (SDR family) member 7 (DHRS7), and integrin subunit beta 2 (ITGB2). The qPCR analysis indeed demonstrated that the mRNA levels of five DEGs were increased (EMP2, gp130, MMP1, PLAU and TNSF15) and that three DEGs were decreased (AKR7A2, DHRS7 and ITGB2) upon golgin-97 knockdown, supporting the reliability of this gene profile analysis (Fig. 3a). Next, these differentially expressed genes associated with golgin-97 expression were then subjected to KEGG pathway analysis, and several significant biological pathways were identified, including transcriptional misregulation in cancer, cytokine-cytokine receptor interaction, mitogen-activated protein kinase (MAPK) signaling pathway, and PI3K-Akt signaling pathway (Table 1). Notably, 31 upregulated genes functioning in KEGG pathways, including positive regulation of multicellular organismal process, response to wounding, positive regulation of biological process, positive regulation of cellular process, and response to stress were transcriptionally regulated by the master gene NF-κB p65/RelA (Fig. 3b). This analysis also revealed that several extracellular molecules (cytokines, chemokines, metalloproteases and growth factors) as well as membrane proteins (cytokine receptors, toll-like receptors and adhesion molecules) were indeed shown in this pathway network (Fig. 3b). To further confirm that NF-κB p65/RelA binding sites are more enriched in the 428 upregulated genes upon golgin-97 knockdown, a promoter analysis of these genes was performed using the TRRUST v2 database [27]. This transcription factor (TF) enrichment analysis showed that NF-κB p65/RelA indeed acted as a major TF that responded to golgin-97 knockdown (Table 2). Accordingly, we proposed that the NF-κB signaling pathway and its related secreted molecules are involved in golgin-97-mediated cell migration/invasion. To confirm this, cell migration/invasion abilities of NF-κB inhibitor-treated control and golgin-97-knockdown MDA-MB-231 cells were examined. As shown in Fig. 3c, treatment with the NF-κB inhibitor noticeably abolished the golgin-97 knockdown-induced migration ability. To further examine whether golgin-97 knockdown can trigger the expression and secretion of NF-κB downstream effectors and promote cell motility, we treated recipient cells with conditioned medium (CM) from control or golgin-97-knockdown cells and examined cells by migration and invasion assays. In Fig. 3d and e, significantly more cells migrated or invaded when cell cultures were treated with CM from golgin-97-knockdown cells than when cultures were treated with CM from control cells. These data demonstrated that the secreted proteins regulated by NF-κB contributed to golgin-97-mediated cell motility. Collectively, these results suggest that golgin-97 regulates cell migration/invasion abilities through modulating NF-κB activity in cancer cells.

**Table 1:** Significant KEGG pathways deduced from the differentially expressed genes in golgin-97-knockdown cells compared with control cells

Term	Count	*p*-value	Genes
Rheumatoid arthritis	14	3.2E-05	ATP6V1C1, ICAM1, CCL3, CCL2, CCL20, IL18, TEK, IL1B, ITGB2, LTB, MMP1, TGFB1, IL1A, TGFB2
Transcriptional misregulation in cancer	17	8.3E-04	MEF2C, PLAT, SLC45A3, LMO2, BAIAP3, RXRB, WHSC1, NR4A3, HIST1H3B, HIST1H3D, HIST1H3F, RUNX1, CD14, PLAU, HIST1H3H, CDK14, HIST1H3I
Cytokine-cytokine receptor interaction	20	1.6E-03	CSF3, CCL3, CCL2, TNFSF4, IL6ST, TNFRSF25, IL18, TNFSF15, IL7R, TGFB1, TGFB2, TNFRSF9, CXCL14, CCL20, IL1RAP, CXCR6, IL1B, EPOR, LTB, IL1A
Malaria	8	2.7E-03	CSF3, ICAM1, CCL2, IL18, IL1B, ITGB2, TGFB1, TGFB2
MAPK signaling pathway	19	1.1E-02	MEF2C, LAMTOR3, FGFR3, TAOK1, CACNB1, PPM1A, TGFB1, FLNA, TGFB2, PLA2G4A, MAPT, RASGRP2, IL1B, PRKACB, CD14, MAP3K12, IL1A, RASA2, AKT2
Legionellosis	7	1.8E-02	IL18, CASP7, IL1B, ITGB2, TLR5, SAR1B, CD14
Alcoholism	14	2.1E-02	HIST1H2AB, HIST1H2AC, HIST1H4L, HDAC3, GNB1, HIST2H2BF, HIST2H2AC, HIST1H3B, HAT1, HIST1H3D, HIST1H2AK, HIST1H3F, HIST1H3H, HIST1H3I
PI3K-Akt signaling pathway	22	2.8E-02	CSF3, YWHAZ, FGFR3, PGF, TNC, ITGA2, IL7R, RPTOR, BRCA1, LAMA1, YWHAH, GNB1, ITGB8, COL6A3, TEK, COL6A2, COL6A1, EPOR, PRKAA1, EIF4E2, AKT2, F2R
Systemic lupus erythematosus	11	3.6E-02	HIST1H2AB, HIST1H2AC, HIST1H4L, HIST2H2BF, HIST2H2AC, HIST1H3B, HIST1H3D, HIST1H2AK, HIST1H3F, HIST1H3H, HIST1H3I
Inflammatory bowel disease (IBD)	7	3.8E-02	IL18, IL1B, TLR5, TGFB1, IL1A, STAT3, TGFB2
Salmonella infection	8	4.3E-02	CCL3, IL18, WASF2, IL1B, TLR5, FLNA, CD14, IL1A
Hematopoietic cell lineage	8	4.8E-02	CSF3, GP1BB, IL1B, ITGA2, EPOR, IL7R, CD14, IL1A

Underlines indicate genes transcriptionally regulated by NF-κB

**Table 2:** Transcription factor enrichment analysis based on the 428 upregulated genes upon golgin-97 knockdown

TF	Count	*p* value*	Genes
SP1	19	1.18E-03	LAMA1, ICAM1, CCL2, CD14, TNC, EPOR, FBLN1, ALOX5, DNMT1, TGFB1, FGFR3, PLAU, IRF5, OXTR, CXCL14, PLAT, PADI1, ATP2A2, F2R
RELA	11	2.30E-02	IL1B, ICAM1, PLAU, CCL2, TNC, ALOX5, CD58, MMP1, IL1A, OXTR, TNFRSF9
NFKB1	11	2.35E-02	MMP1, IL1B, TNC, TNFRSF9, PLAU, ALOX5, IL1A, ICAM1, TGFB1, CD58, CCL2
JUN	9	1.70E-03	IL1B, PLAT, CCL2, ITGB8, IL1A, TNC, OXTR, PLAU, MMP1
SP3	8	1.11E-03	FBLN1, F2R, LAMA1, PADI1, PLAT, CD14, ATP2A2, FGFR3
STAT3	7	1.68E-02	DNMT1, PGF, F2R, TGFB1, ICAM1, MMP1, CCL2
NFKBIA	4	3.42E-04	ICAM1, IL1B, MMP1, CD58
ATF2	4	2.63E-03	PLAT, TGFB2, ITGB8, PLAU
TWIST1	4	3.67E-03	F2R, ICAM1, MMP1, NR2F1
KLF4	4	5.45E-03	LAMA1, CD14, IL1B, CDKN1C
FOS	4	2.03E-02	OXTR, MMP1, IL1A, PLAU
PPARG	4	3.26E-02	ATP2A2, ANGPTL4, MMP1, ICAM1
REL	3	7.21E-03	CCL2, IL1B, ICAM1
TWIST2	3	1.03E-02	ICAM1, F2R, NR2F1
RARA	3	1.15E-02	ICAM1, RPTOR, PLAU
HDAC2	3	1.28E-02	ALOX5, CCL2, DNMT1
SMAD3	3	1.87E-02	TNC, ANGPTL4, TGFB1
ETS2	3	2.03E-02	MMP1, ICAM1, TNC
ATF4	3	2.57E-02	PLAU, CCL2, DISC1
GATA3	3	3.19E-02	TEK, MMP1, EPOR
IRF3	2	2.99E-02	CCL2, IRF5

*Asterisk indicates a significant enrichment with a *p*-value< 0.05 as determined by Fisher’s exact test

### Golgin-97 knockdown induces NF-κB activation by reducing IκBα levels

**Fig. 4:**
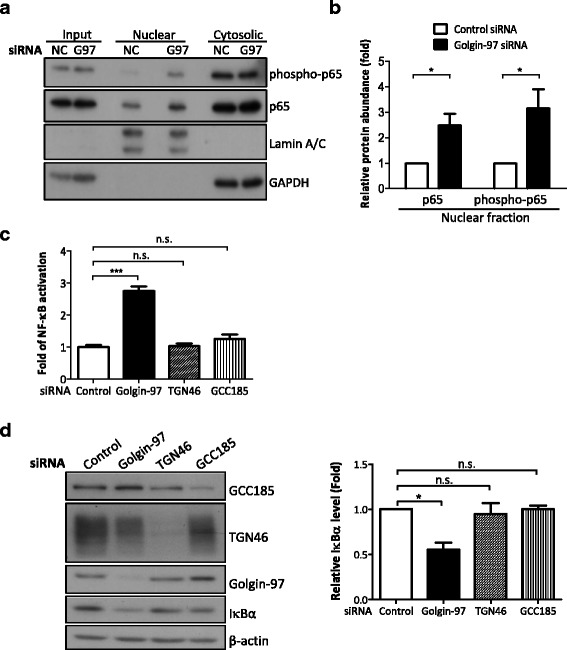
Golgin-97 knockdown induces NF-κB activation by reducing IκBα levels. **a** Western blot analysis of nuclear entry of phospho-p65 in control (NC) or golgin-97 (G97)-knockdown cells. GAPDH and Lamin A/C were used as controls for cytosolic and nuclear fractions, respectively. **b** Quantification analysis of nuclear p65 and phospho-p65 acquired from western blot analysis. **c** NF-κB activation determined by luciferase reporter assay. **d** IκBα protein levels were reduced in golgin-97-knockdown cells. Actin was used as the internal control. Quantitative results are presented as the means±SEM from three independent experiments. *p < 0.05. ***p < 0.001. n.s., no significance

To confirm the role of golgin-97 in modulating NF-κB activity, we performed subcellular fractionation to examine nuclear entry of active NF-κB (phospho-p65) in control and golgin-97-knockdown cells. Figure 4a and b show that the levels of p65 and phospho-p65 in nuclear fractions from golgin-97-knockdown cells were higher than those detected in fractions from control cells. Next, NF-κB activities were determined using an NF-κB luciferase reporter assay, and as expected, the NF-κB activity in golgin-97-knockdown cells was higher than that in control cells (Fig. 4c). Notably, knockdown of TGN-localized integral membrane glycoprotein TGN46 or TGN-localized GRIP domain protein GCC185 did not cause significant NF-κB activation, suggesting a specific role for golgin-97 in regulating NF-κB activity (Fig. 4c). Considering that IκBα, a member of the IκB family of inhibitor proteins, directly interacts with NF-κB and in turn controls NF-κB activation [30–33], we also examined IκBα levels in golgin-97-knockdown cells. As expected, IκBα protein levels were significantly decreased in golgin-97-knockdown cells but not in TGN46- or GCC185-knockdown cells (Fig. 4d). However, we also observed that TGN46 protein levels were reduced in golgin-97-knockdown cells, which might have been due to impaired plasma membrane-TGN recycling of TGN46 (Fig. 4d). Taken together, these data suggest that depletion of golgin-97 specifically activates NF-κB activity in vivo.

### Loss of Golgi integrity is not involved in the golgin-97 knockdown-induced NF-κB activation

**Fig. 5:**
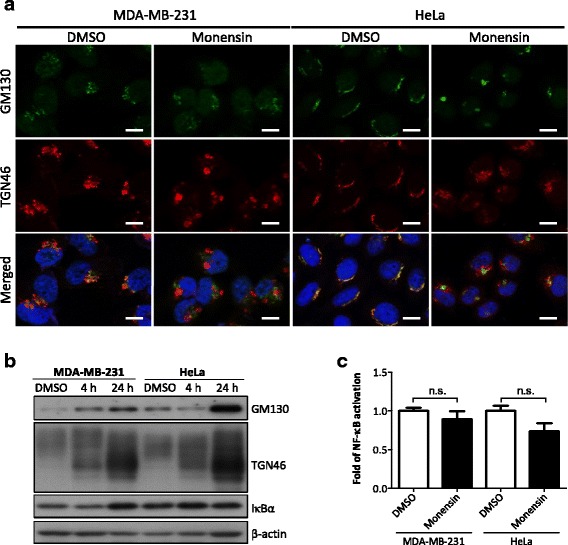
Loss of Golgi integrity is not involved in the golgin-97 knockdown-induced NF-κB activation. **a** Representative images of Golgi markers in monensin-treated MDA-MB-231 or HeLa cells. Scale bars, 10 μm. **b** Western blot analysis of Golgi markers in monensin-treated MDA-MB-231 or HeLa cells. Actin was used as the internal control. **c** Luciferase reporter assay of monensin-treated MDA-MB-231 or HeLa cells. Relative levels of NF-κB activation are presented as the mean ± SEM from three independent experiments. n.s., no significance

It is well documented that GRIP domain proteins such as golgin-97 and GCC185 are required for maintaining Golgi integrity [21, 34]. Thus, we proposed that Golgi fragmentation caused by golgin-97 knockdown might induce Golgi stress and subsequent NF-κB activation. To test this possibility, we first examined the effects of a Golgi stress inducer and ionophore monensin on the regulation of NF-κB activation. In line with the previous studies [35, 36], monensin caused severe morphological changes in the Golgi apparatus such that swollen vesicles emerged near the nucleus were observed in monensin-treated HeLa cells (Additional file 3: Figure S2). An immunofluorescence assay (IFA) revealed that the TGN46 signal was dispersed from the TGN and also found in peripheral swollen vesicles, whereas the *cis*-Golgi GM130 signal was reduced or aggregated upon monensin treatment for 4 h in HeLa or MDA-MB-231 cells, respectively (Fig. 5a). Moreover, western blot analysis demonstrated a significant molecular weight shift of TGN46 from high to low in a time-dependent manner, whereas the GM130 levels were variable (Fig. 5b). The bands of TGN46 at low molecular weight correspond to the immature TGN46 with defective glycosylation, in particular in sialylation, as reported recently [37]. These results indicated that monensin acts as an effective Golgi-disturbing agent in the cells used in this study. However, surprisingly, results from the NF-κB luciferase reporter assay showed that monensin treatment did not induce significant NF-κB activation in these two cell lines (Fig. 5c). Altogether, these results suggest that Golgi fragmentation might be not responsible for the NF-κB activation in golgin-97-knockdown cells.

### Golgin-97 functions as an NF-κB suppressor regardless of its subcellular localization

**Fig. 6:**
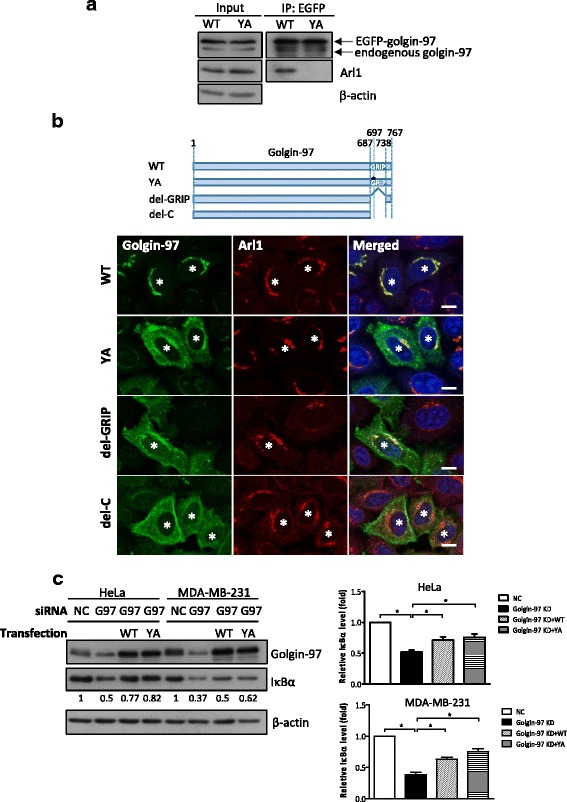
The interaction of golgin-97 with Arl1 is not necessary for its function in the modulation of NF-κB activity. **a** Golgin-97 WT, but not the YA mutant, interacts with Arl1. HeLa cells expressing WT EGFP-golgin-97 or the YA mutant were extracted and subsequently immunoprecipitated as described in the Materials and Methods. Input (30 μg) and IP products were analyzed by western blot with anti-golgin-97, anti-Arl1 or anti-actin antibodies. **b** Representative images of the subcellular localization of Arl1 (red) and golgin-97 WT, YA, del-GRIP and del-C mutants (green) in HeLa cells. Schematic diagram presents the golgin-97 truncations and mutations used in this study. Scale bars, 10 μm. Asterisks indicate golgin-97-overexpressing cells. **c** Western blot analysis of IκBα expression in the WT golgin-97 or the YA mutant-rescued golgin-97-knockdown HeLa or MDA-MB-231 cells. Actin was used as the internal control. Quantitative results are presented as the means±SEM from three independent experiments. *p < 0.05

**Fig. 7:**
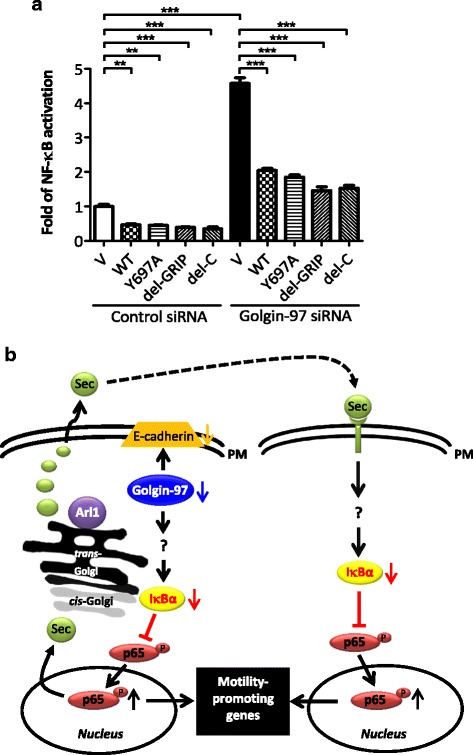
Golgin-97 functions as an NF-κB suppressor regardless of its subcellular localization. **a** Luciferase reporter assay of golgin-97 WT, YA, del-GRIP or del-C mutant-rescued golgin-97-knockdown MDA-MB-231 cells. Quantitative results are presented as the means±SEM from three independent experiments. **p < 0.01. ***p < 0.001. **b** Proposed model of golgin-97 in modulating cancer invasiveness. Sec, secreted molecule. PM, plasma membrane

Golgin-97 is recruited to the Golgi by interacting with GTP-bound Arl1 through its GRIP domain [38]. To test whether golgin-97 knockdown-induced NF-κB activation relies on this interaction between golgin-97 and Arl1, we generated and expressed the golgin-97 siRNA-1-resistant WT or Arl1-binding-defective mutant (YA) construct in golgin-97-knockdown cells. As expected, immunoprecipitation (IP) analysis demonstrated that golgin-97 WT, but not the YA mutant, interacts with Arl1 (Fig. 6a). The IFA also showed that golgin-97 co-localized with Arl1 on the Golgi, most of the signal for the YA mutant was dispersed throughout the cytoplasm and did not co-localize with Arl1 (Fig. 6b). Moreover, reduced IκBα levels were also found to be restored in YA mutant-expressing cells (Fig. 6c), suggesting that both the TGN-bound and cytosolic pools of golgin-97 are involved in modulating NF-κB activation. We then used a luciferase reporter assay to confirm that overexpression of not only the WT but also the YA mutant significantly suppressed the NF-κB activity induced by golgin-97 knockdown (Fig. 7a). In addition, two golgin-97 mutants with either the deletion of the entire GRIP domain (del-GRIP) or a C-terminus truncation (del-C) were generated and used to confirm that the cytosolic golgin-97 (Fig. 6b) had the ability to rescue the golgin-97-knockdown phenotype (Fig. 7a). Little is known about the function of golgins in regulating signal transduction. Recently, You et al. identified arfaptin-2, one of the TGN golgins, as an IκB kinase β (IKKβ)-interacting protein via yeast two-hybrid screening [39]. In the present study, we examined the interaction between golgin-97 and IKKβ by IP. However, neither WT nor mutated golgin-97 associated with IKKβ (Additional file 3: Figure S3). We then proposed that, under normal physiological conditions, golgin-97 might interact with some unidentified molecule(s) to regulate the protein levels of IκBα and that this would in turn suppress NF-κB activation. However, in cancer cells, golgin-97 downregulation would lead to activation of NF-κB and secretion of cancer-promoting molecules that would in turn enhance cell motility. Consistent with this, dysregulated distribution of E-cadherin was also found to be involved in this processing (Fig. 7b).

## Discussion

Golgins belong to a conserved family of Golgi-associated proteins that are crucial for vesicular trafficking and maintaining Golgi structure and positioning within cells [6, 40]. However, their roles in cancer and tumorigenesis remain unclear. There are at least 11 golgins [6, 40], and the *cis*-Golgi matrix protein GM130 is the best characterized cancer-related golgin. In the past decade, similar to our finding shown in Fig. 1a, studies have revealed the role of GM130 as a potential oncogene and tumor suppressor gene [41]. For example, Chang et al. showed that loss of GM130 decreased angiogenesis and cancer cell invasion in vitro and suppressed tumorigenesis in a lung cancer mouse model [41]. Moreover, Zhao et al. found that GM130 expression positively correlated with pathological differentiation and tumor node metastasis of gastric cancer [42]. However, a suppressive role of GM130 in breast and colorectal cancers was reported by Farhan’s group [43]. The authors identified a GM130-RasGRF-Cdc42 connection in regulating polarity and tumorigenesis and found that silencing GM130 is sufficient to induce E-cadherin downregulation [43]. Moreover, they showed that depletion of GM130 increases cellular velocity and the invasiveness of breast cancer cells, suggesting that GM130 is a tumor suppressor in breast cancer [44]. Taken together, these studies indicate the important roles of golgins in modulating tumorigenesis.

Interestingly, the significance of Golgi fragmentation in cancer biology has recently been explored [45]. Knockdown of *cis*-Golgi proteins golgin-160 and GMAP210 was reported to lead to fragmentation of the Golgi into several mini-stacks and to inhibition of cell migration [46]. GRIP domain proteins such as GCC185 and golgin-97 are well known to be required for maintenance of Golgi structure [16, 34]. However, in this study, we observed increased NF-κB activity in golgin-97-knockdown but not GCC185-knockdown cells (Fig. 4). To further reduce golgin-97 expression, we performed a second RNAi treatment and extended the RNAi treatment time to 96 h (Additional file 3: Figure S4A). Notably, Golgi fragmentation was observed after golgin-97 repeated knockdown for 72 h and 96 h, and GM130 was significantly dispersed from the Golgi ribbon (Additional file 3: Figure S4B). However, the IκBα level was significantly reduced after a single treatment of golgin-97 siRNA for 24 h, and a similar reduction of IκBα levels was observed after a second RNAi treatment for 72 h or 96 h (Additional file 3: Figure S4A), suggesting that NF-κB activation occurred at the early stage of golgin-97 depletion and did not result from Golgi fragmentation. Consistent with these observations, we treated cells with monensin to induce severe Golgi stress and found no significant change in NF-κB activity in monensin-treated cells (Fig. 5c). We also compared our microarray data with a list of monensin-induced genes generated by Yoshida’s group [36] and found no correlation. Thus, we proposed that the NF-κB-mediated cell migration/invasion promoted by loss of golgin-97 would be transient and that this regulation occurs before Golgi fragmentation.

Golgin-245 is the most closely related GRIP-domain tethering protein to golgin-97. Recently, Wong et al. showed that both golgin-97 and golgin-245 capture vesicles via their first 21 residues at the N-terminus, which is necessary and sufficient to nucleate the capture of endosome-to-Golgi carriers [15]. However, in this current study, we propose that the function of golgin-97 in the regulation of NF-κB activity would not rely on its role for vesicle trafficking at TGN. The reasons are as follows: First, three cytosolic golgin-97 mutants (Fig. 6b), comprising golgin-97 YA, golgin-97 del-GRIP, and golgin-97 del-C had the ability to rescue the golgin-97-knockdown phenotypes (Fig. 7a). Second, deletion of the first 21 residues at the N-terminus of golgin-97 (golgin-97 del-N) resulted in the loss of its vesicle capture ability, but this construct still could suppress the increase o NF-κB activity induced by golgin-97 knockdown (Additional file 3: Figure S5). Third, golgin-245 knockdown did not significantly alter NF-κB activity (Additional file 3: Figure S6). These evidences support our view that the regulation of NF-κB activity by golgin-97 is not restricted to its TGN localization and would not rely on its role for vesicle trafficking/recycling.

Different signaling pathways and molecules at the Golgi, including the MAPK pathway, Rho GTPases, cyclin-dependent kinase (CDK) pathway, phosphoinositide signaling, protein kinase D pathway and mTOR signaling, have been proposed to regulate directional cell migration [3]. In the current study, we identified NF-κB activation as a possible factor responsible for golgin-97 knockdown-induced cancer cell motility. We provide several lines of support for a possible function of golgin-97 as an inhibitor of NF-κB signaling pathway: (1) golgin-97 knockdown reduced IκBα protein levels and induced the nuclear entry of active NF-κB (phospho-p65) and subsequent NF-κB activation; (2) treatment of NF-κB inhibitor abolished golgin-97 knockdown-induced cell migration; and (3) golgin-97 overexpression significantly suppressed the NF-κB activity induced by golgin-97 knockdown. Moreover, we found that the inhibitory effects of golgin-97 in NF-κB activation were independent of Golgi integrity. NF-κB is well known to be involved in regulating immune responses and inflammation; however, growing evidence has supported its crucial role in oncogenesis. NF-κB regulates the expression of genes that play key roles in cancer development and progression such as proliferation, migration and apoptosis. Aberrant or constitutive NF-κB activation has been detected in many human malignancies [47, 48]. In breast cancer cells, NF-κB was reported to repress E-cadherin expression, enhance EMT, and be involved in regulating EMT genes [49, 50]. In colon cancer cell migration, Jung et al. found that activin exploits NF-κB activity to induce mouse double minute 2 homolog (MDM2) activity, leading to p21 degradation in a PI3K-dependent manner [51]. However, the mechanism by which golgin-97 affects NF-κB activity in cancer cells is unknown. Recently, You et al. identified arfaptin-2, a BAR-domain protein and an effector of Arl1, in the TGN as an IKKβ-specific binding partner. They showed that arfaptin-2 overexpression inhibited TNF-α-stimulated NF-κB signaling, whereas arfaptin-2 knockdown enhanced NF-κB activity [39]. In the current study, we also examined whether golgin-97 interacts with IKKβ. The results of the IP experiments indicated that golgin-97 did not co-immunoprecipitate with IKKβ and vice versa (Additional file 3: Figure S3). Therefore, further investigations regarding how and when golgin-97 is involved in regulating the NF-κB pathway will be required to address these issues.

Accumulation of misfolded proteins in the endoplasmic reticulum (ER) was previously reported to cause stress that subsequently leads to NF-κB activation and protects cells from apoptosis [52]. Nakajima et al. found a putative ER stress-response element on the promoter of mitochondrial ubiquitin ligase activator of NF-κB (MULAN) and showed that MULAN expression was upregulated by ER stress. Their findings suggested that MULAN is an E3 ligase that regulates NF-κB activation to protect cells from ER stress-induced apoptosis [52]. Being a tethering factor and a scaffold molecule for secretion at the TGN, we cannot rule out the possibility that the protein missorting or unfolding caused by golgin-97 knockdown might induce ER stress and subsequent NF-κB activation. Thus, further validation will be required to address these issues.

## Conclusion

In summary, we report for the first time that golgin-97 functions as a tumor suppressor. We also provided new insights into the role of golgin-97 in suppressing NF-κB signaling as well as cancer cell invasiveness. Accordingly, our future work will employ a proteomic approach to systemically identify distinct protein complexes for TGN-bound and cytosolic golgin-97 to uncover unappreciated modulators and regulatory networks of cancer cell migration. These studies will provide a basis for the development of new therapeutic strategies against cancer cell metastasis.

## Additional files

Additional file 1: **Table S1.** The siRNA sequences used in this study. (XLS 24 kb)

Additional file 2: **Table S2.** The primer sequences used in this study. (XLS 27 kb)

Additional file 3: **Figure S1.** Golgin-97 knockdown reduces surface-bound E-cadherin in breast cancer cells. **Figure S2.** Monensin causes morphological changes in the Golgi apparatus. **Figure S3.** Golgin-97 does not interact with IKKβ. **Figure S4.** Effects of repeated and long-term treatment of golgin-97 siRNA on IκBα levels and Golgi integrity in HeLa cells. **Figure S5.** Effects of the Golgin-97 del-N mutant on NF-κB activity. **Figure S6.** Golgin-245 knockdown has no significant effects on NF-κB activity in breast cancer cells. (PDF 2242 kb)

## Abbreviations

Arl1: Arf-like protein 1
CM: Conditioned medium
DMSO: Dimethyl sulfoxide
EGFP: Enhanced green fluorescent protein
EMT: Epithelial-to-mesenchymal transition
ER: Endoplasmic reticulum
GRIP: **g**olgin-97/**R**anBP2α/**I**mh1/**p**230/golgin-245
IKKβ: IκB kinase β
MAPK: Mitogen-activated protein kinase
MULAN: Mitochondrial ubiquitin ligase activator of NF-κB
qPCR: Quantitative PCR
RT-PCR: Reverse transcription polymerase chain reaction
SEM: Mean of standard deviation
TF: Transcription factor
TGN: *Trans*-Golgi network
TNF: Tumor necrosis factor
WT: Wild type
