# Supplementary material for: Golgi tethering factor golgin-97 suppresses breast cancer cell invasiveness by modulating NF-κB activity
Source: Cell Commun Signal. 2018 Apr 27;16:19. doi: 10.1186/s12964-018-0230-5 (PMC5923015; doi:10.1186/s12964-018-0230-5)
Supplement: Supplementary file 3 — Figure S1. Golgin-97 knockdown reduces surface-bound E-cadherin in breast cancer cells. Figure S2. Monensin causes morphological changes in the Golgi apparatus. Figure S3. Golgin-97 does not interact with IKKβ. Figure S4. Effects of repeated and long-term treatment of golgin-97 siRNA on IκBα levels and Golgi integrity in HeLa cells. Figure S5. Effects of the Golgin-97 del-N mutant on NF-κB activity. Figure S6. Golgin-245 knockdown has no significant effects on NF-κB activity in breast cancer cells. (PDF 2242 kb) [file 12964_2018_230_MOESM3_ESM.pdf]

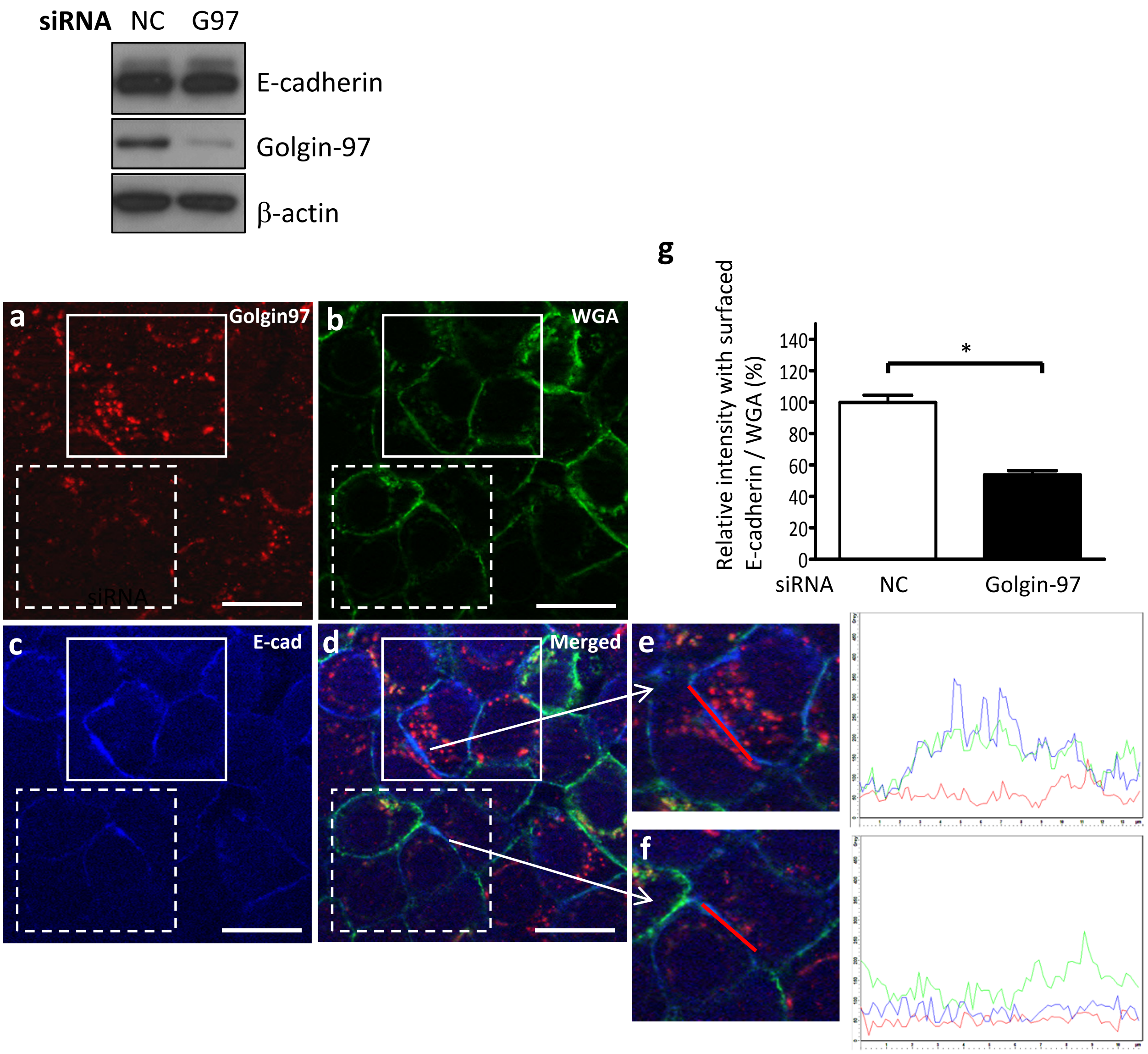

Fig. S1. Golgin-97 knockdown reduces surface-bound E-cadherin in breast cancer cells. MDA-MB-231 cells were transfected with control (NC) or golgin-97 (G97)-specific siRNA oligos, as indicated. After 48 h, the cells were subjected to western blot analysis with anti-golgin-97 and anti-E-cadherin antibodies. Actin was used as the internal control. Meanwhile, control and golgin-97-knockdown cells were seeded onto coverslips and immunostained with antibodies against golgin-97, E-cadherin and fluorescently tagged wheat germ agglutinin (WGA) lectin, as indicated (a-d). Golgin-97 (red) and E-cadherin (blue) intensities and localizations were determined via AxioVS40 software (e and f). Relative intensities of cells (n=30) with surface-bound E-cadherin/WGA were analyzed and calculated (g). The results are presented as the mean±SEM; *$p<0.05$ indicates significance using the Mann-Whitney test.

Figure S1

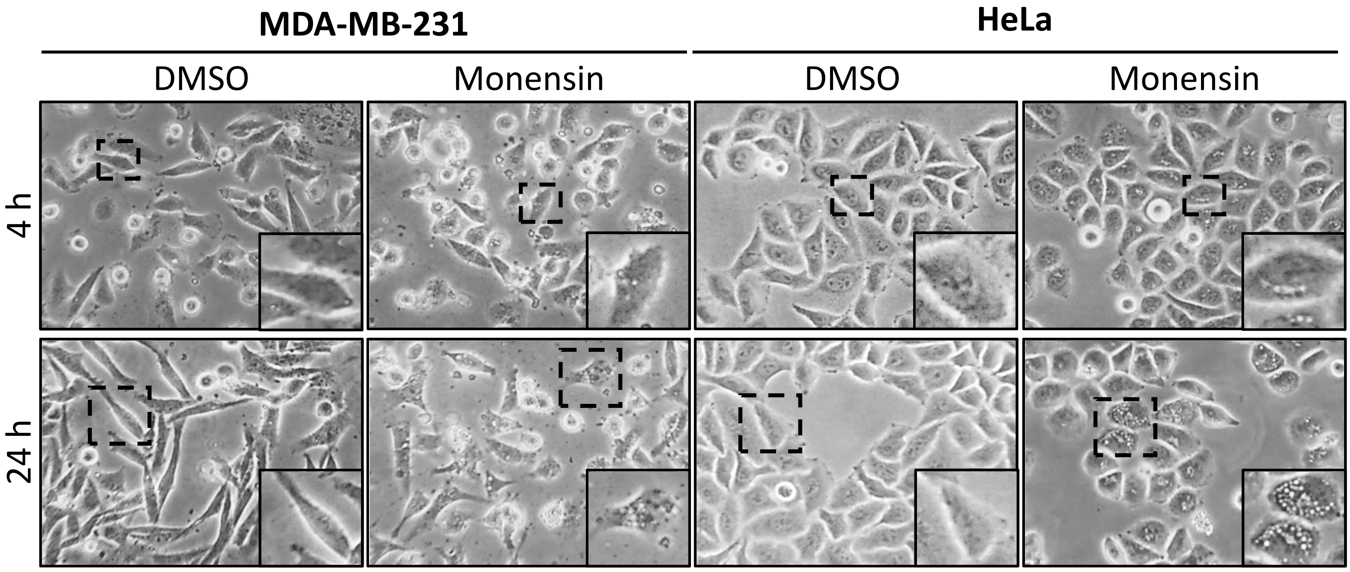

Fig. S2. Monensin causes morphological changes in the Golgi apparatus. MDA-MB-231 or HeLa cells on cover glasses were treated with DMSO or 10 mM monensin for 4 h and 24 h, as indicated. Cells were examined by phase-contrast microscopy.

Figure S2

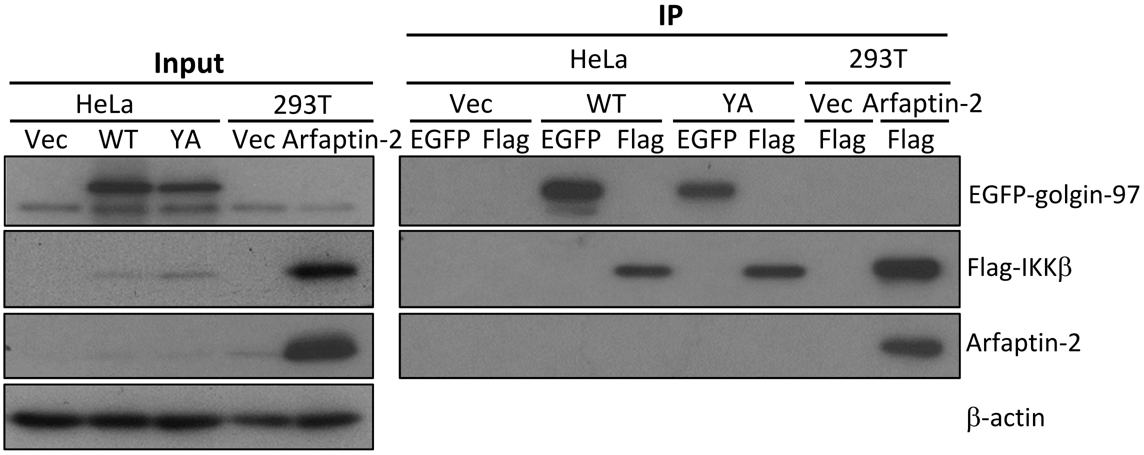

Fig. S3. Golgin-97 does not interact with IKKβ. HeLa cells expressing Flag-IKKβ together with the EGFP vector (Vec), EGFP-golgin-97 WT or YA were collected and immunoprecipitated using anti-GFP nanobodies or an anti-Flag M2 antibody coupled to magnetic beads. Input (30 μg) and IP products were resolved by 7.5% SDS-PAGE followed by western blot with anti-golgin-97, anti-Flag or anti-actin antibodies. The flag-IKKβ construct was a kind gift from Dr. Jong-Ik Hwang, Korea University (You, Park et al. 2015). IP products using lysates of 293T cells expressing vector (Vec) or arfaptin-2 served as a positive control for the IP protocol and reagents.

Figure S3

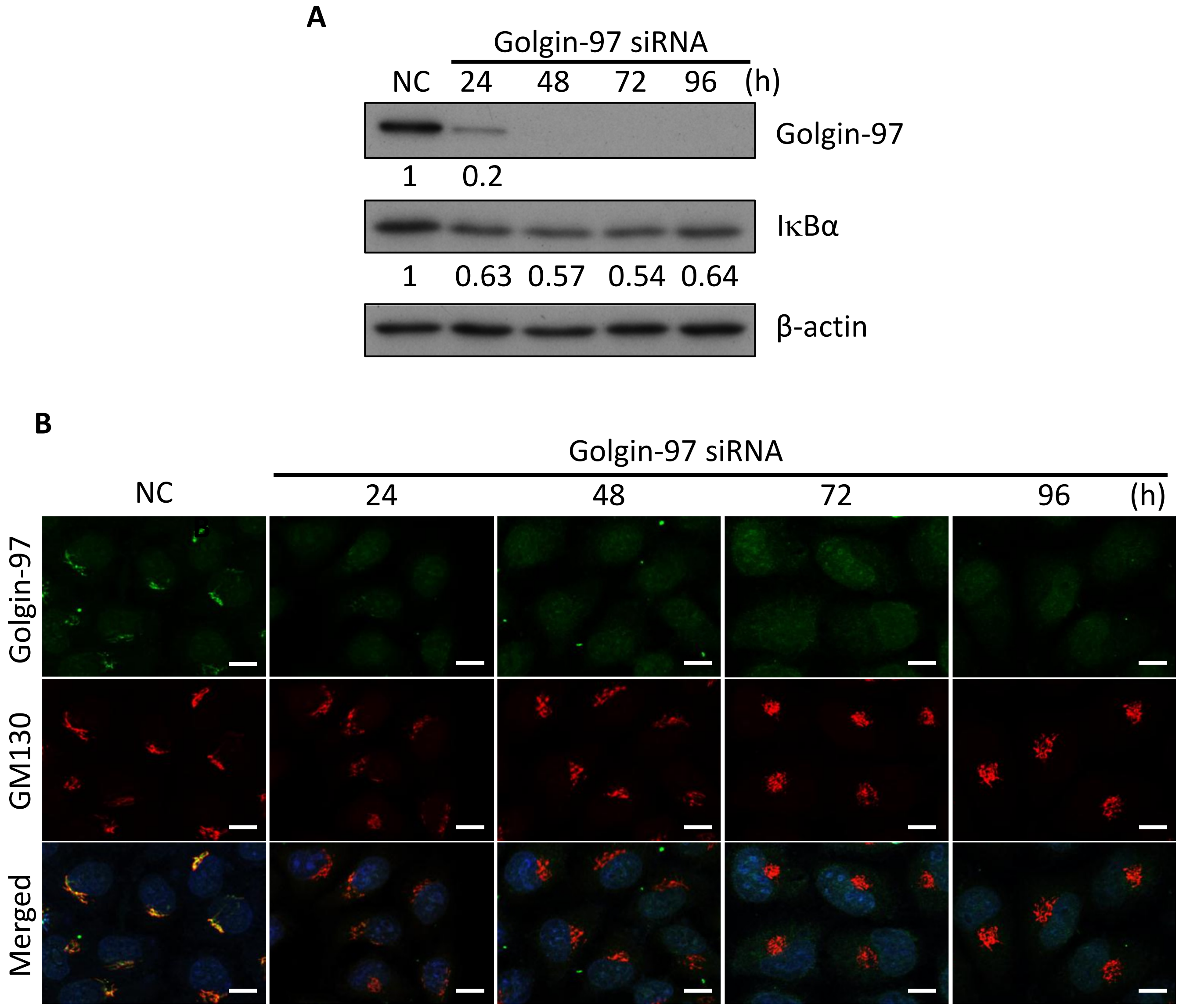

Fig. S4. Effects of repeated and long-term treatment of golgin-97 siRNA on IκBα levels and Golgi integrity in HeLa cells. (A) HeLa cells were transfected with control (NC) or golgin-97-specific siRNA oligos, as indicated. After 48 h, cells were then transfected a second time with golgin-97-specific siRNA oligos for a further 24 h and 48 h. Lysates of siRNA oligo-treated for different times were harvested and subjected to western blot analysis with anti-golgin-97 and anti-IκBα antibodies. Actin was used as the internal control. (B) Subcellular localizations of golgin-97 and GM130 in golgin-97-knockdown HeLa cells were analyzed by IFA with anti-golgin-97 (green) and anti-Arl1 (red) antibodies, as indicated. DNA was stained with Hoechst 33258 (blue). Scale bars, 10 μm. Images were acquired using a Zeiss ApoTome fluorescence microscope and AxioVision Rel 4.8 software.

Figure S4

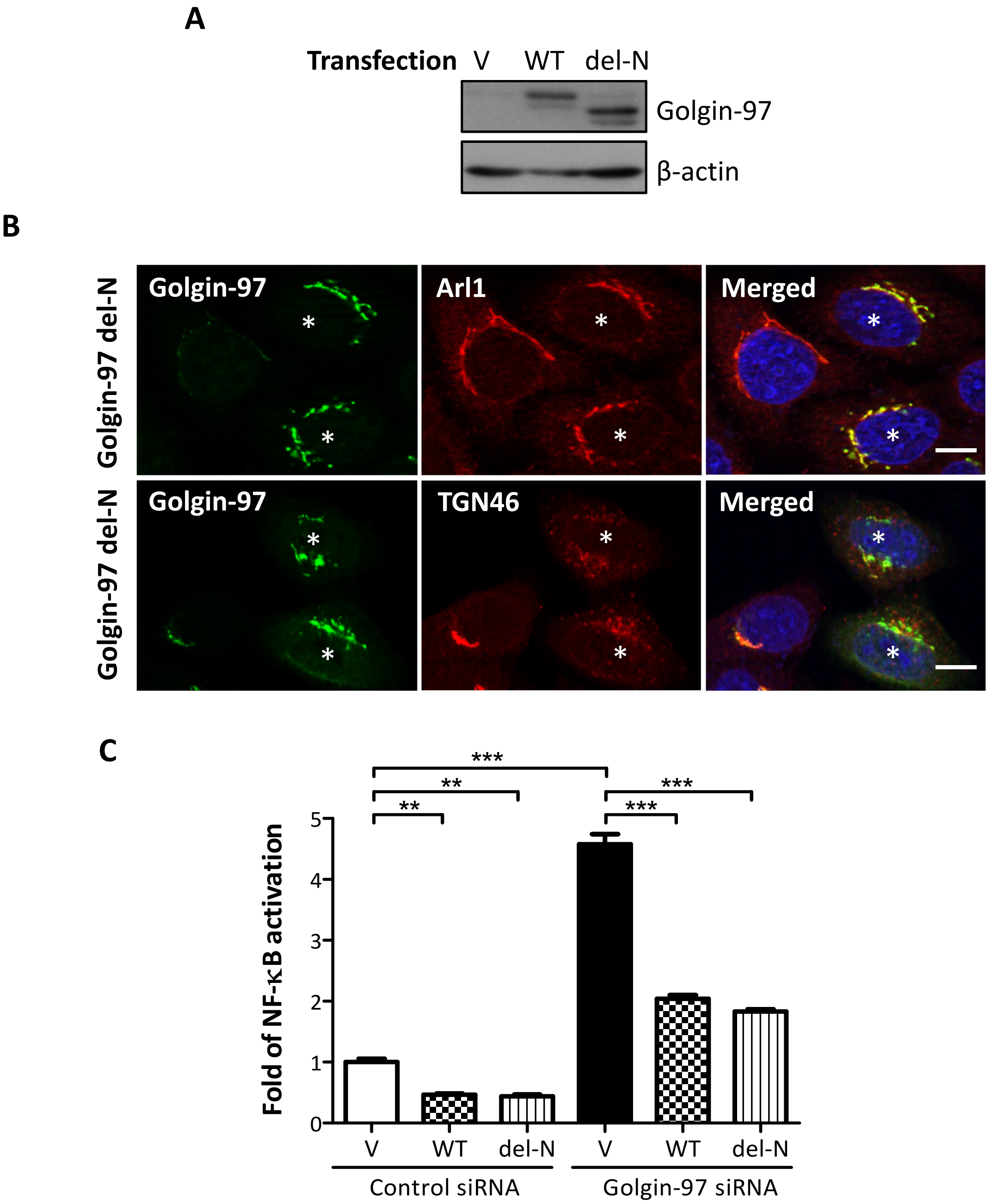

Fig. S5. Effects of the golgin-97 del-N mutant on NF-κB activity. (A) HeLa cells expressing the golgin-97 del-N mutant (deleted amino acids 1-21) were extracted and subjected to SDS-PAGE followed by western blot with anti-golgin-97 or anti-actin antibodies. (B) Golgin-97 del-N mutant localizes at TGN, whereas the TGN46 (in vesicles captured by golgin-97) is partially dispersed into the cytoplasm in golgin-97 del-N-expressing cells. HeLa cells on cover glasses were transfected with the golgin-97 del-N mutant. After 24 h, the cells were subjected to IFA with anti-golgin-97, anti-Arl1, and anti-TGN-46 antibodies, as indicated. DNA was stained with Hoechst 33258 (blue). Scale bars, 10 μm. Images were acquired using a Zeiss ApoTome fluorescence microscope and AxioVision Rel 4.8 software. Asterisks indicate golgin-97 del-N mutant-overexpressing cells. (C) Overexpression of the golgin-97 del-N mutant significantly suppresses NF-κB activity induced by golgin-97 knockdown. MDA-MB-231 control or golgin-97-knockdown cells expressing the golgin-97 del-N mutant were extracted and subjected to luciferase reporter assays. Relative levels of NF-κB activation are presented as the mean±SEM from three independent experiments. **$p<0.01$. ***$p<0.001$.

Figure S5

A

B

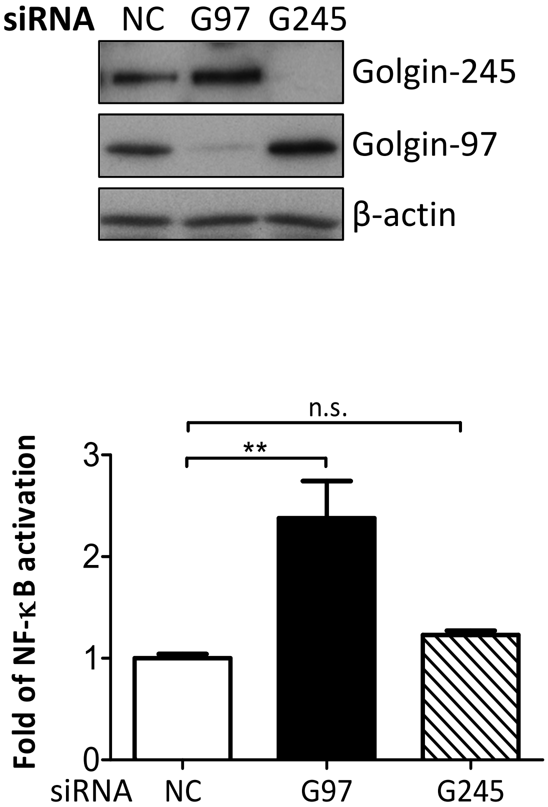

Fig. S6. Golgin-245 knockdown has no significant effects on NF-κB activity in breast cancer cells. MDA-MB-231 cells were transfected with control (NC), golgin-97 (G97)- or golgin-245 (G245)-specific siRNA oligos, as indicated. After 48 h, the cells were harvested and analyzed by western blot (A) and luciferase reporter assays (B). Relative levels of NF-kB activation are presented as the mean±SEM from three independent experiments. $^{**}p<0.01$. n.s., no significance.

Figure S6
